# Moving Toward a Consensus: Comparison of the 2023 ESH and 2017 ACC/AHA Hypertension Guidelines

**DOI:** 10.1016/j.jacadv.2024.101230

**Published:** 2024-08-28

**Authors:** Prasantha L. Vemu, Eugene Yang, Joseph E. Ebinger

**Affiliations:** a Department of Medicine, University of Washington School of Medicine, Seattle, Washington, USA; b Division of Cardiology, University of Washington School of Medicine, Seattle, Washington, USA; c Department of Cardiology, Smidt Heart Institute, Cedars-Sinai Medical Center, Los Angeles, California, USA

**Keywords:** blood pressure, cardiovascular disease, guidelines, hypertension

**Figure:**
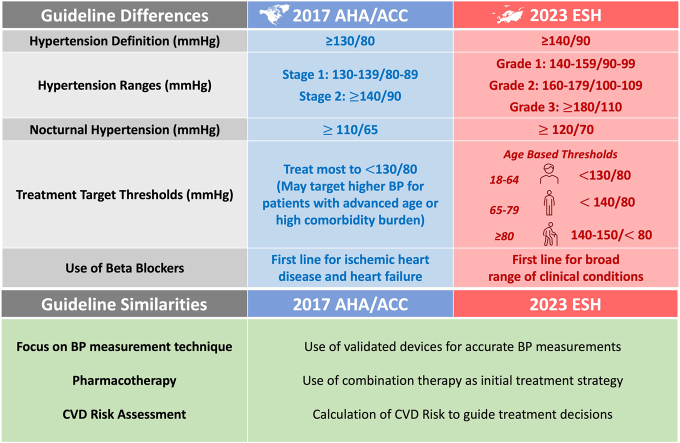

Hypertension is a primary modifiable risk factor for coronary artery disease (CAD), heart failure, stroke, and chronic kidney disease (CKD).^1^ The 2017 American College of Cardiology/American Heart Association (ACC/AHA) Guidelines for the Prevention, Detection, Evaluation, and Management of High Blood Pressure in Adults and the recently published 2023 European Society of Hypertension (ESH) guidelines for the management of arterial hypertension are two primary sources on best practices for hypertension diagnosis and treatment.^2^^,^^3^This analysis reviews major updates in the 2023 ESH BP guideline and highlights key similarities and differences with the 2017 ACC/AHA blood pressure (BP) guideline.

Compared to the 2018 European Society of Cardiology/ESH BP guideline, changes in the 2023 ESH guideline were relatively minor.^4^ Importantly, the 2023 ESH guideline is more concordant with the 2017 ACC/AHA guideline in several key respects (Figure 1). Both guidelines emphasize the use of standardized measurement protocols to ensure the accuracy of BP measurements. This includes utilizing validated, cuffed devices to obtain office BP measurements across multiple clinic visits. The ESH guideline recommends against the use of cuffless devices, expressing concerns about reliance of cuffless devices on incompletely validated predictive technologies to estimate BP, absence of standardized validation protocols to ensure accuracy of these devices, and need for periodic calibration with cuffed devices.^5^^,^^6^ Both guidelines agree that home blood pressure monitoring (HBPM) or ambulatory blood pressure monitoring (ABPM) should be used prior to diagnosing hypertension since these methods capture the dynamic BP fluctuations that occur during daily life compared to a static snapshot that is obtained by office-based measurements. HBPM uses commercially available validated, cuffed devices to self-monitor BPs at home, while ABPM devices are automated to record BPs at specified timed intervals during a 24- to 48-hour period. Both guidelines highlight the importance of using HBPM and ABPM to detect masked and white-coat hypertension.

**Figure 1:**
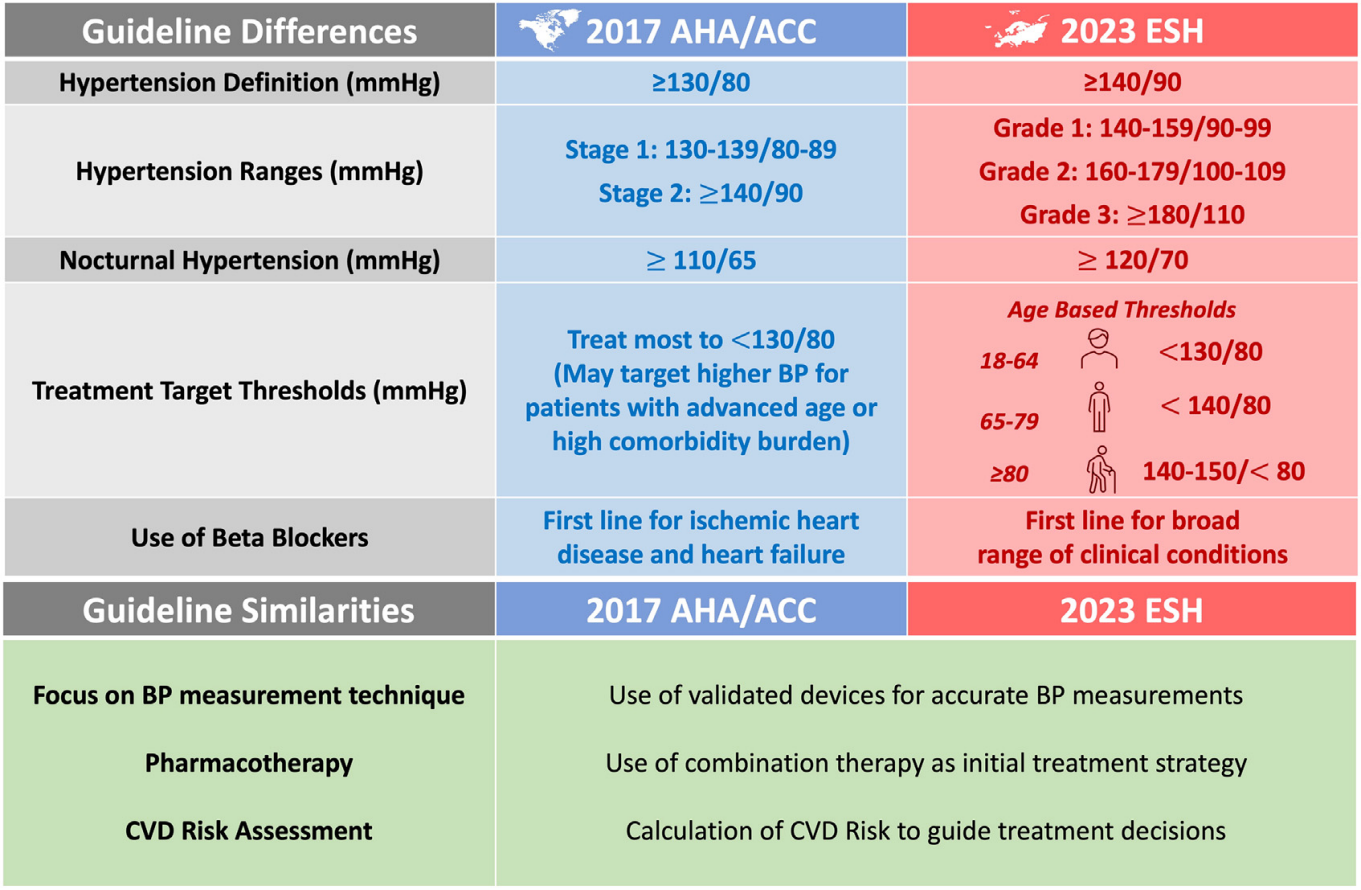
**Similarities and Differences Between the 2017 ACC/AHA and 2023 ESH Hypertension Guidelines** ACC/AHA = American College of Cardiology/American Heart Association; BP = blood pressure; CVD = cardiovascular disease; ESH = European Society of Hypertension.

The importance of atherosclerotic cardiovascular disease (ASCVD) risk stratification tools in guiding treatment decisions is stressed by both guidelines. The 2023 ESH update emphasizes the use of the updated Systematic Coronary Risk Evaluation 2 (SCORE2) risk prediction model that includes nonfatal ASCVD events and is more aligned with the pooled cohort equation recommended by U.S. guidelines.^7^ SCORE2 is derived from large cohort data of healthy Europeans and predicts ASCVD risk for individuals aged 40 to 69 years. The U.S. guideline recommends the use of pooled cohort equation to estimate the 10-year risk of fatal and nonfatal ASCVD events for adults between the ages of 40 and 79 years.^2^ The ESH guideline also recommends a separate risk assessment tool, SCORE2-Older Persons (SCORE2-OP) for adults ≥70 years, derived from a large, Norwegian cohort.^8^ Although these tools can guide treatment decisions, it is important to note that these models were created using derivation cohorts that may not be generalizable to all patients, potentially impacting risk estimates.^9^

The central role of lifestyle interventions to treat and prevent hypertension is promoted by both guidelines. These recommendations include emphasis on optimal weight control, heart healthy diet with sodium restriction, structured physical activity, smoking cessation, and moderation of alcohol consumption. The ACC/AHA guideline also recommends increased potassium intake for patients with hypertension or elevated BP without CKD, which is a new addition to the current 2023 ESH guideline (Class of Recommendation [COR] 1, Level of Evidence [LOE]: B). The European guideline also explicitly discusses environmental risk enhancers, such as noise and air pollution, as important risk factors for hypertension which are not included in the U.S. guideline.

Both guidelines provide similar recommendations on thresholds for initiation of antihypertensive pharmacotherapy. The ACC/AHA guideline recommends initiation of antihypertensive agents along with lifestyle modifications for patients diagnosed with “stage 1 hypertension” (130-139 mm Hg/80-89 mm Hg) and clinical ASCVD, defined as CAD, stroke, transient ischemic attack, or peripheral artery disease, or an estimated 10-year ASCVD risk >10%. ESH also recommends treatment initiation for patients with BP measurements in the “high-normal” range (systolic BP [SBP] ≥130mm Hg or diastolic blood pressure ≥80 mm Hg) who also have a high CVD risk due to existing CVD, especially CAD. Both recommend that patients aged 18 to 79 years (ESH) and adults ≥18 years (ACC/AHA) with SBP ≥140 mm Hg and/or diastolic blood pressure ≥90 mm Hg (“stage 2 hypertension per ACC/AHA and “grade 1” per ESH) should be prescribed antihypertensive therapy regardless of ASCVD risk or existing clinical CVD. ESH specifies that for adults aged ≥80 years, pharmacotherapy should be initiated when SBP >160 mm Hg (COR I, LOE: B).

ACC/AHA and ESH guidelines recommend initial treatment with antihypertensive agents from at least 1 of 4 major classes: angiotensin-converting enzyme inhibitors, angiotensin-receptor blockers, thiazide or thiazide-like diuretics, and calcium channel blockers. The ESH guideline continues to recommend beta-blockers as an optional first-line therapy for several CV and many non-CV conditions, including atrial fibrillation, aortic dissection, diabetes, and chronic obstructive pulmonary disease, while the American guideline only recommends them for patients with ischemic heart disease or heart failure.^10^ The ESH guideline points to several randomized control trials and meta-analyses that show beta-blockers significantly reduce major CVD events when compared to placebo.^4^

Initiation of combination therapy with medications from different classes is also prominent in both guidelines. The ACC/AHA guideline recommends an initial two-drug regimen for patients with "stage 2 hypertension” and BP > 20/10 mm Hg above target, and for Black patients (COR I, LOE: C-EO), while ESH recommends initial therapy with a two-drug combination regimen for most patients with hypertension (COR I, LOE: A). ESH strongly advocates for single-pill combination therapy to improve medication adherence and achieve BP control, while the U.S. guideline recommends either single-pill combination or multi-pill combinations.

BP grade classifications and definitions did not change between the 2018 European Society of Cardiology/ESH guideline and updated 2023 ESH guideline. As a result, significant differences in BP classification and definition of hypertension between guidelines remain, with ACC/AHA using “stages” and ESH using “grades” for BP categorization (Figure 1). ESH defines hypertension as BP ≥140/90 mm Hg, while ACC/AHA uses a threshold of ≥130/80 mm Hg. ESH classifies systolic BP between 130 and 139 mm Hg or diastolic ranges between 85 and 89 mm Hg as “high-normal” BP, while ACC/AHA guideline defines this as “stage 1” hypertension.

Recommendations on BP treatment targets also differ. For most patients, the ACC/AHA guideline recommends treating to a BP target of <130/80 mm Hg. The ESH, on the other hand, recommends tailoring treatment based on age, clinical comorbidities, and patient-specific tolerance. For adults between ages 18 and 64 years, the ESH guideline recommends a BP target <130/80 mm Hg, similar to what is recommended by the ACC/AHA guideline. However, the ESH guideline recommends higher targets for older patients. For adults aged 65 to 79 years, ESH recommends targeting BP <140/80 mm Hg, but notes that if tolerated by the patient, a target of <130/80 mm Hg should be used. The threshold for very old patients (≥80 years old) is even higher, targeting SBP between 140 and 150 mmHg. For special populations including hypertensive patients with CKD, the ESH guideline recommends treatment to a higher BP target of <140/90 mm Hg compared to the recommended ACC/AHA BP target of <130/80 mm Hg. For other comorbidities including diabetes, CAD, cerebrovascular disease, both guidelines recommend the same BP target of <130/80 mm Hg. While significant differences still exist between the guidelines, for high-risk patients and individuals between ages 18 and 79 years old, there is consensus to achieve BP goal <130/80 mm Hg.

A major difference between guidelines is the recommendation of renal denervation therapy for hypertension treatment by the 2023 ESH guideline (COR II, LOE: B), which is absent from the ACC/AHA guideline. Renal denervation therapy is a device-based treatment intended for patients with uncontrolled resistant hypertension (after excluding secondary hypertension) as either an additional or alternative therapy to increasing medication burden.

The updated 2023 ESH BP guideline is a comprehensive resource for hypertension management. The changes highlighted in this review demonstrate that the recommendations of the U.S. and European guidelines continue to harmonize. Both emphasize proper BP measurement technique, advise use of ASCVD risk assessment tools for treatment decisions, and recommend similar classes of antihypertensive therapies. The guidelines continue to differ in BP classification, hypertension definition, BP targets for older adults, and beta-blocker and device-based treatment strategies. Overall, there is strong agreement that for most people, especially those at highest ASCVD risk, BP < 130/80 mm Hg should be encouraged.

## Funding support and author disclosures

Dr Yang is supported by the UW Medicine Asian Health Initiative and the Carl and Renée Behnke Endowed Professorship for Asian Health; has served on the Advisory Board of Chroma, Measure Labs, Mineralys, Qure.ai, Sky Labs, TenPoint7; has received consultation fees from Genentech; honoraria from American College of Cardiology; and research grants from Microsoft Research. Dr Ebinger is supported by grants from the National Institutes of Health (K23-HL153888; R21HL156132-01); and has received consultation fees from Edwards Lifesciences and Rubicon Founders. Dr Vemu has reported that she has no relationships relevant to the contents of this paper to disclose.
